# Supplementary figures and images for: Dis3L2 regulates cell proliferation and tissue growth through a conserved mechanism
Source: PLoS Genet. 2020 Dec 28;16(12):e1009297. doi: 10.1371/journal.pgen.1009297 (PMC7793271; doi:10.1371/journal.pgen.1009297)

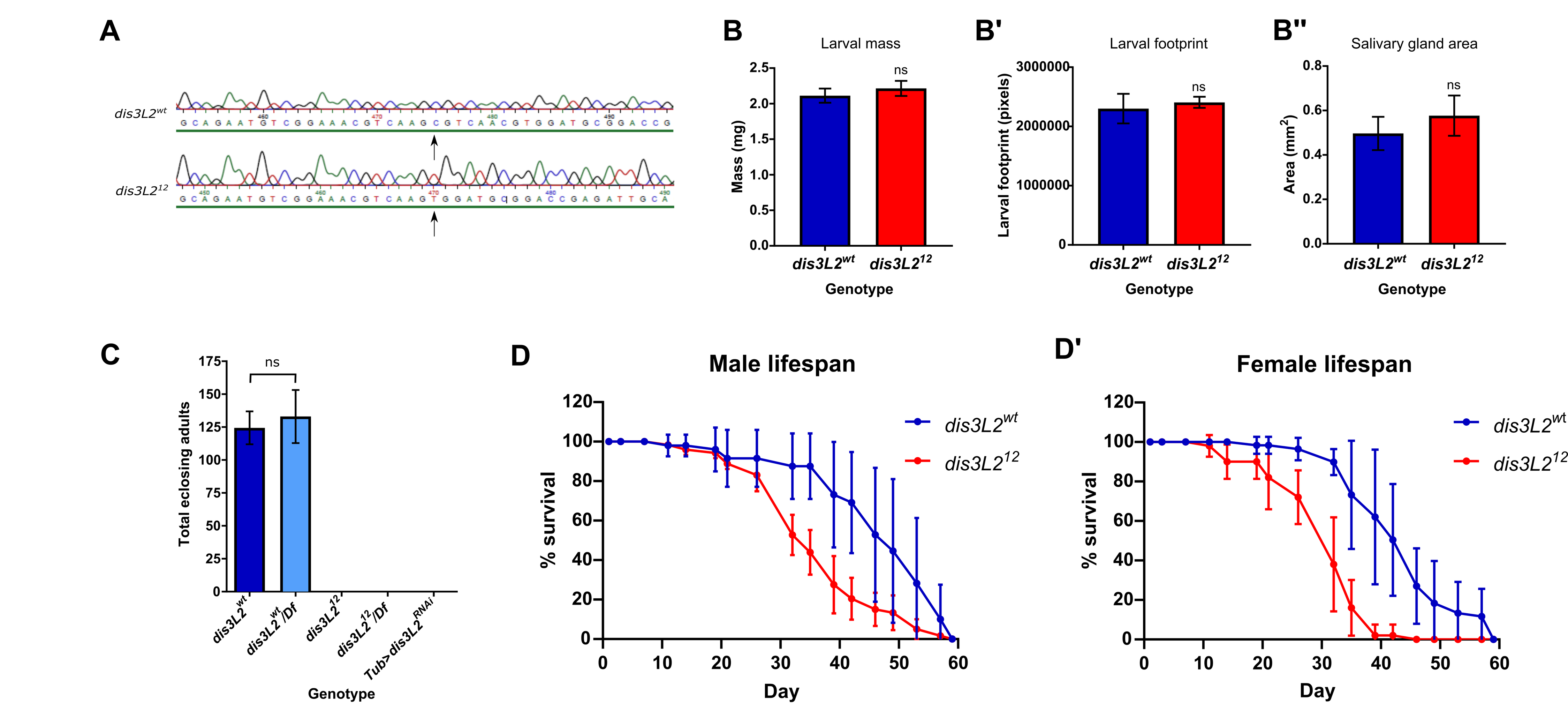

Supplement: S1 Fig — A) DNA sequencing of the control dis3L2wt and mutant dis3L212 lines where the arrow denotes the site of mutation. B/B’/B”) The mass (B), footprint (B’) or salivary gland area (B”) of dis3L212 120hr old L3 larvae are not significantly different from dis3L2wt controls. n = 16–31, error bars represent 95% CI, ns = p>0.05. C) Male flies lacking Dis3L2 are infertile. In all crosses no progeny was observed for those using homozygous (dis3L212), hemizygous (dis3L212/Df) or dis3L2 knockdown (Tub>dis3L2RNAi) males. Error bars represent 95% CI, ns = p>0.05. For all crosses males of each genotype were crossed to virgin dis3L2wt females. D/D’) dis3L212 mutant males (D) and females (D’) have a reduced lifespan compared to dis3L2wt isogenic controls. n = 47–57, error bars represent 95% CI with significant differences demonstrated by the lack of overlapping error bars. (TIF) [file pgen.1009297.s001.tif]

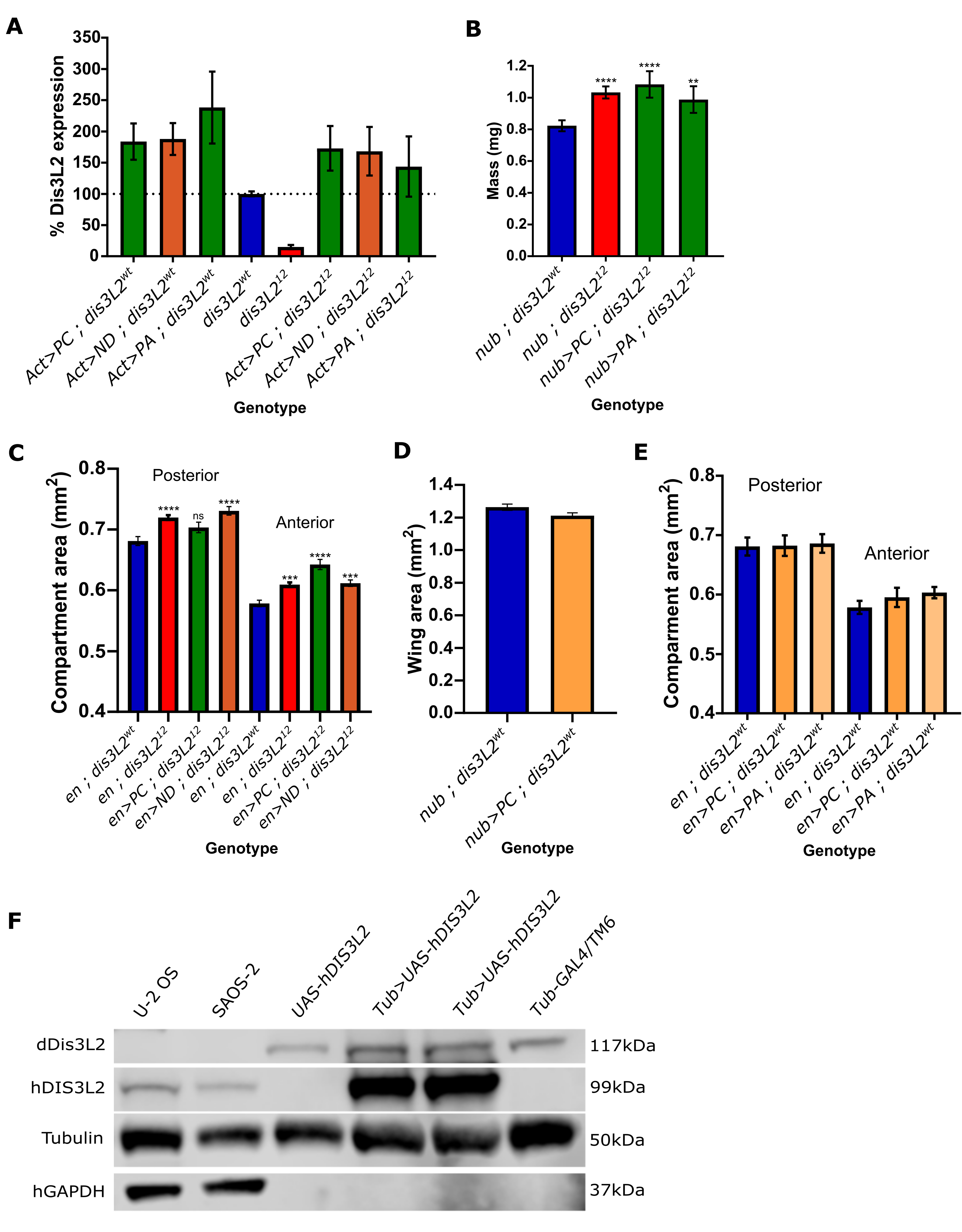

Supplement: S2 Fig — A) Quantification of Western blots performed on 3, 1-day old, female flies of the demonstrated genotypes. n = 4–10, error bars represent SEM. B) Whilst specific re-expression of Dis3L2 in the wing (with nub-GAL4) rescues wing overgrowth it does not rescue organism overgrowth. n = 10–44, error bars represent 95% CI, ** = p<0.01, **** = p<0.0001. C) Re-expression of wild-type Dis3L2 (PC) but not catalytically dead Dis3L2 (ND) in the posterior compartment of the wing/wing imaginal disc results in a specific rescue of the posterior area of the wing whilst the anterior area remains significantly larger. n = 24–44, error bars represent 95% CI, ns = p>0.05, *** = p = <0.001, **** = p = <0.0001. D/E) Mild overexpression of Dis3L2 using the rescue UAS-Dis3L2PA/PC constructs does not have a major effect on wing area when driven by nub-GAL4 (D) or en-GAL4 (E). n = 16–47, error bars represent 95% CI. F) Human DIS3L2 is successfully expressed in Drosophila from the UAS-hDIS3L2 construct. Two human osteosarcoma cells lines were used as positive controls (U-2 OS and SAOS-2). Protein lysate was prepared from 1x10^6^ cells or 4, 1-day old, adult females. hDIS3L2 is observed specifically in the human cells and female flies where UAS-hDIS3L2 had been driven by Tub-GAL4 at 25°C (Tub>UAS-hDIS3L2). No product was observed in the parental controls (UAS-hDIS3L2 and Tub-GAL4/TM6), confirming no ‘leaky’ expression from the UAS-hDIS3L2 line. Tubulin used as a loading control for all samples as the antibody detects both human and Drosophila protein. hGAPDH used as an additional loading control specifically for the human cell line samples. (TIF) [file pgen.1009297.s002.tif]

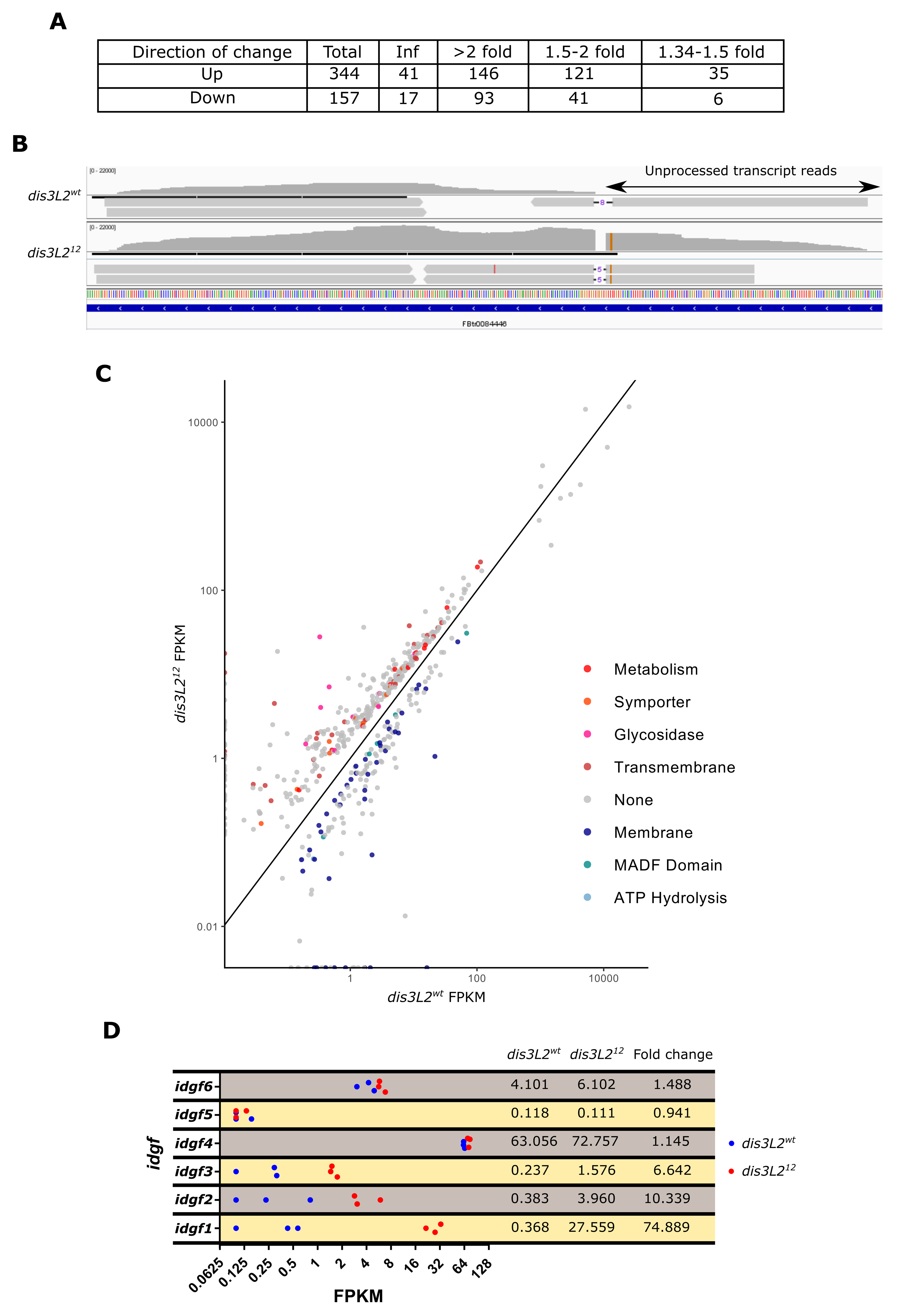

Supplement: S3 Fig — A) Summary of the number of transcripts showing up- and downregulation in dis3L212 wing imaginal discs. A fold change cut off of >1.34 was selected as this was the smallest change deemed significant by Cuffdiff. Inf change represents transcripts that were only detected in a single condition. B) Integrative Genomics Viewer screenshot showing accumulation of unprocessed RNaseMRP:RNA transcripts in dis3L212 tissues. C) Scatter plot of misregulated genes coloured by significant gene ontology categories. "None" represents genes that belonged to a category that was not significantly enriched. D) Strip plots showing replicate FPKM values for each of the idgf family in dis3L2wt and dis3L212 wing discs. Only idgf1, idgf2 and idgf3 show changes in expression. (TIF) [file pgen.1009297.s003.tif]

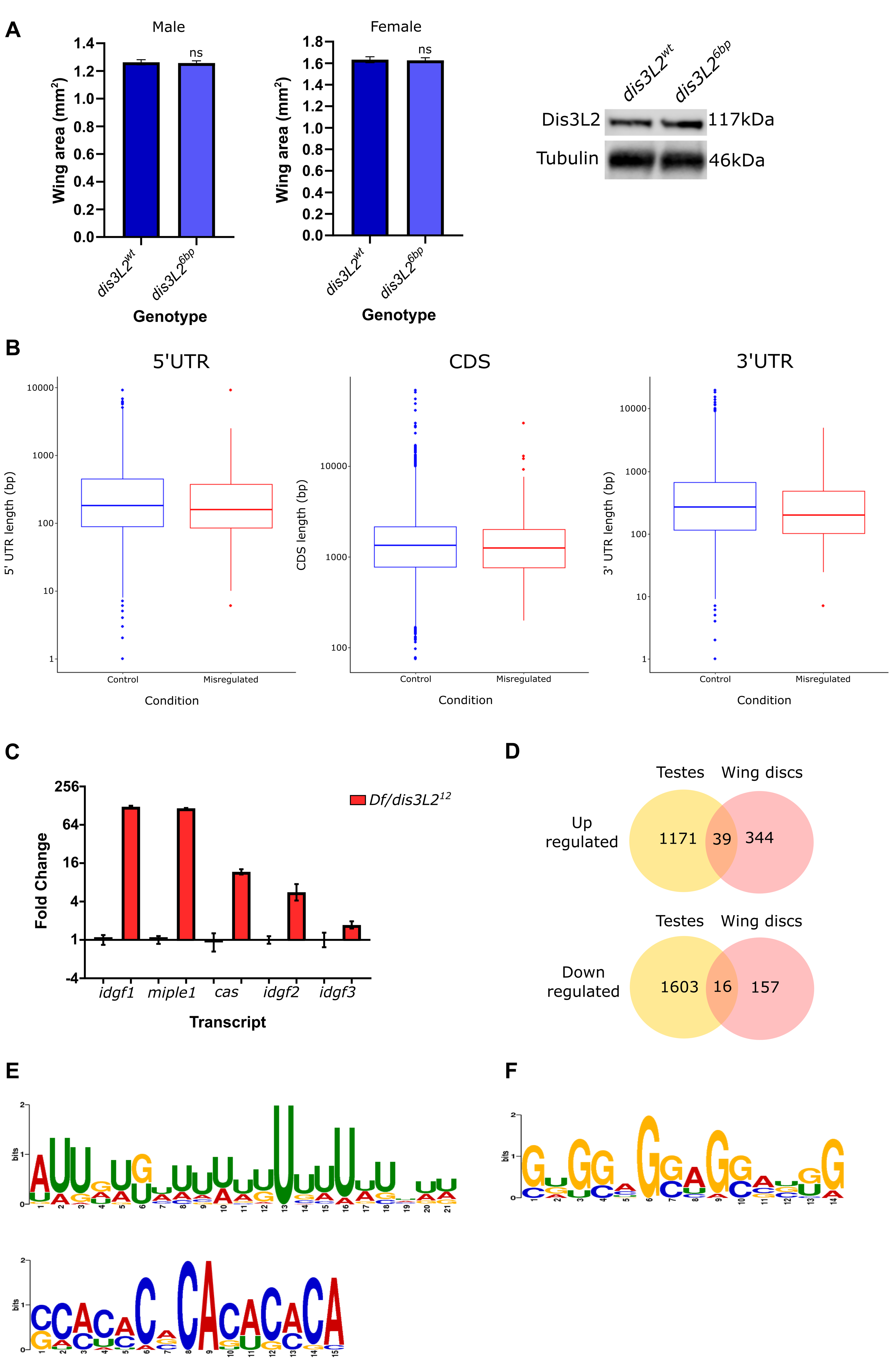

Supplement: S4 Fig — A) The 6nt deletion in the control stock used for RNA-sequencing does not affect Dis3L2 protein expression or wing area in male or female flies. n = 19–33, error bars represent 95% CI, ns = p<0.05. B) Transcripts misexpressed in dis3L212 wing imaginal discs have significantly shorter 3’ UTRs (446nt vs 598nt, Welch 2 sample t-test p = 2.078e-05). 5’ UTRs (337nt vs 365nt) and the coding sequence (CDS, 1712nt vs 1837nt) show no difference in length between transcripts. Median and upper and lower quartile are represented by horizontal lines with maximum and minimum values shown vertically. C) All validated mRNAs show significant increases in expression in dis3L212 hemizygote wing imaginal discs compared to dis3L2wt wing discs. n = 3–6, error bars represent SEM, p<0.05 for all. D) 10% of transcripts misexpressed in dis3L212 wing discs are also misexpressed in dis3L2 mutant testes [11]. E) MEME analysis identifies U-rich and CA-rich motifs are significantly enriched in likely Dis3L2 targets. U-rich: E-value = 5.2e-6, found in 40.6% of submitted sequences. CA-rich: E-value = 0.0012, found in 14.1% of submitted sequences. F) A G-rich motif present in 23.1% of control sequences (sequences that show no change and do not co-precipitate with Dis3L2 [13]) is absent in Dis3L2 target sequences. E-value = 0.016. (TIF) [file pgen.1009297.s004.tif]

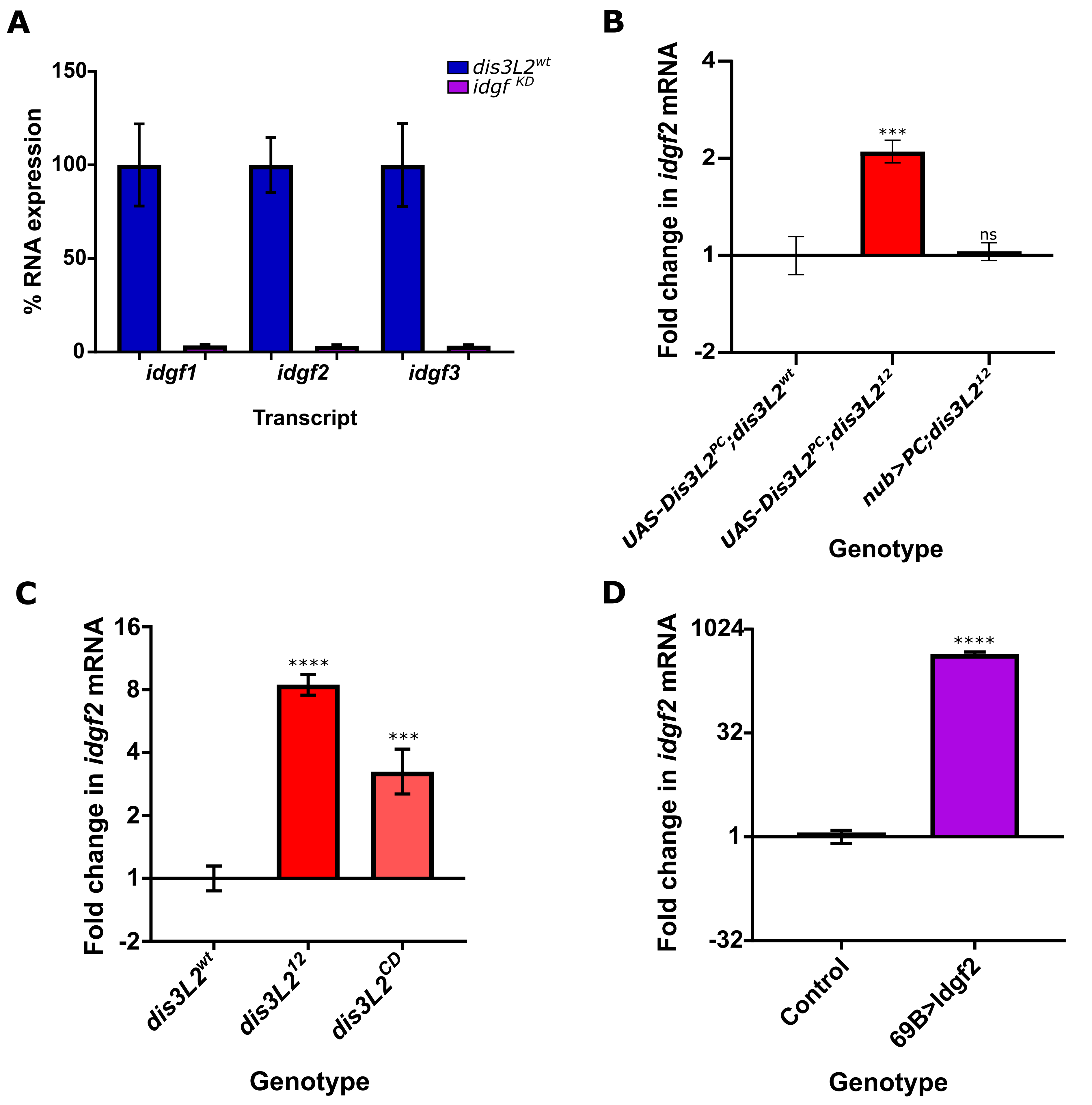

Supplement: S5 Fig — A) Ubiquitous knockdown of idgf1, idgf2 and idgf3 by driving specific UAS-RNAi constructs with Tub-GAL4 results in >90% knockdown for all targets. n = 3, p<0.0004 for all, error bars represent SEM. B) Re-expression of Dis3L2 in dis3L212 wing imaginal discs (nub>PC; dis3L212) results in a reduction of idgf2 mRNA to a level not significantly different from dis3L2wt tissues. n = 5–6, error bars represent SEM, *** = p<0.001, ns = p = 0.4748. C) idgf2 mRNA is significantly increased in expression in the wing imaginal discs of an independent line carrying a CRISPR generated catalytic dead mutation in the endogenous dis3L2 locus (dis3L2CD)[13]. n = 4–6, error bars represent SEM, ****p<0.0001, ***p = 0.0006. D) Driving UAS-Idgf2 with 69B-GAL4 results in a significant increase in idgf2 mRNA in the wing imaginal disc (UAS-idgf2/+; 69B-GAL4/+) compared to controls. Controls include both parental lines. n = 6, 95% CI, **** = p<0.0001. (TIF) [file pgen.1009297.s005.tif]

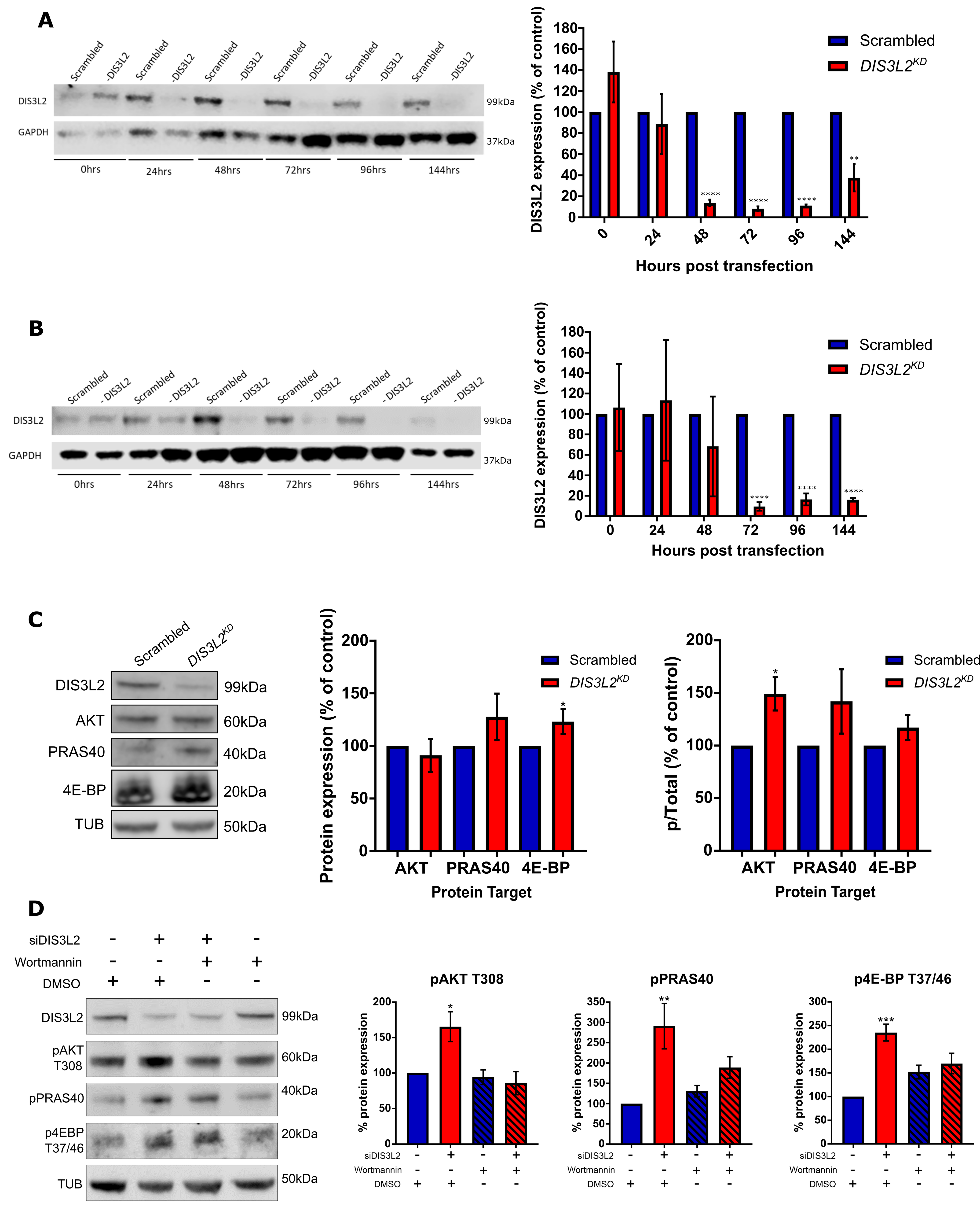

Supplement: S6 Fig — A) Knockdown of DIS3L2 in human kidney HEK-293T cells is observed 48 hours after transfection and is retained until at least 144hrs post-transfection. Maximal knockdown is observed 72 hours post transfection. n = 4, error bars represent SEM, **** = p<0.0001, ** = p<0.01. B) Knockdown of DIS3L2 in human osteosarcoma U-2 OS cells is observed 48 hours after transfection and is retained until at least 144hrs post-transfection. Maximal knockdown is observed 72 hours post transfection. n = 4, error bars represent SEM, **** = p<0.0001. C) Representative image and quantification of Western blots assessing total protein levels of AKT, PRAS40 and 4E-BP in DIS3L2 knockdown or scrambled control HEK-293T cells 72hrs post transfection. n = 7, error bars represent SEM, * = p = 0.00379. Also shown is phosphorylated protein levels normalised to total protein levels. n = 7, error bars represent SEM, * = p = 0.0214. PRAS40 and 4E-BP also show a trend towards more phosphorylated protein but this is not statistically significant (p>0.05). D) Wortmannin treatment reduces pAKT (T308) pPRAS40 and p4E-BP (T37/26) signal in DIS3L2 Knockdown HEK-293T cells 48hrs post transfection. Representative images and quantification of Western blots in DIS3L2KD or Scrambled control HEK-293T cells treated with either DMSO or 250nM Wortmannin. n = 4, error bars represent SEM, * = p<0.05, ** = p<0.01, *** = p<0.001. (TIF) [file pgen.1009297.s006.tif]

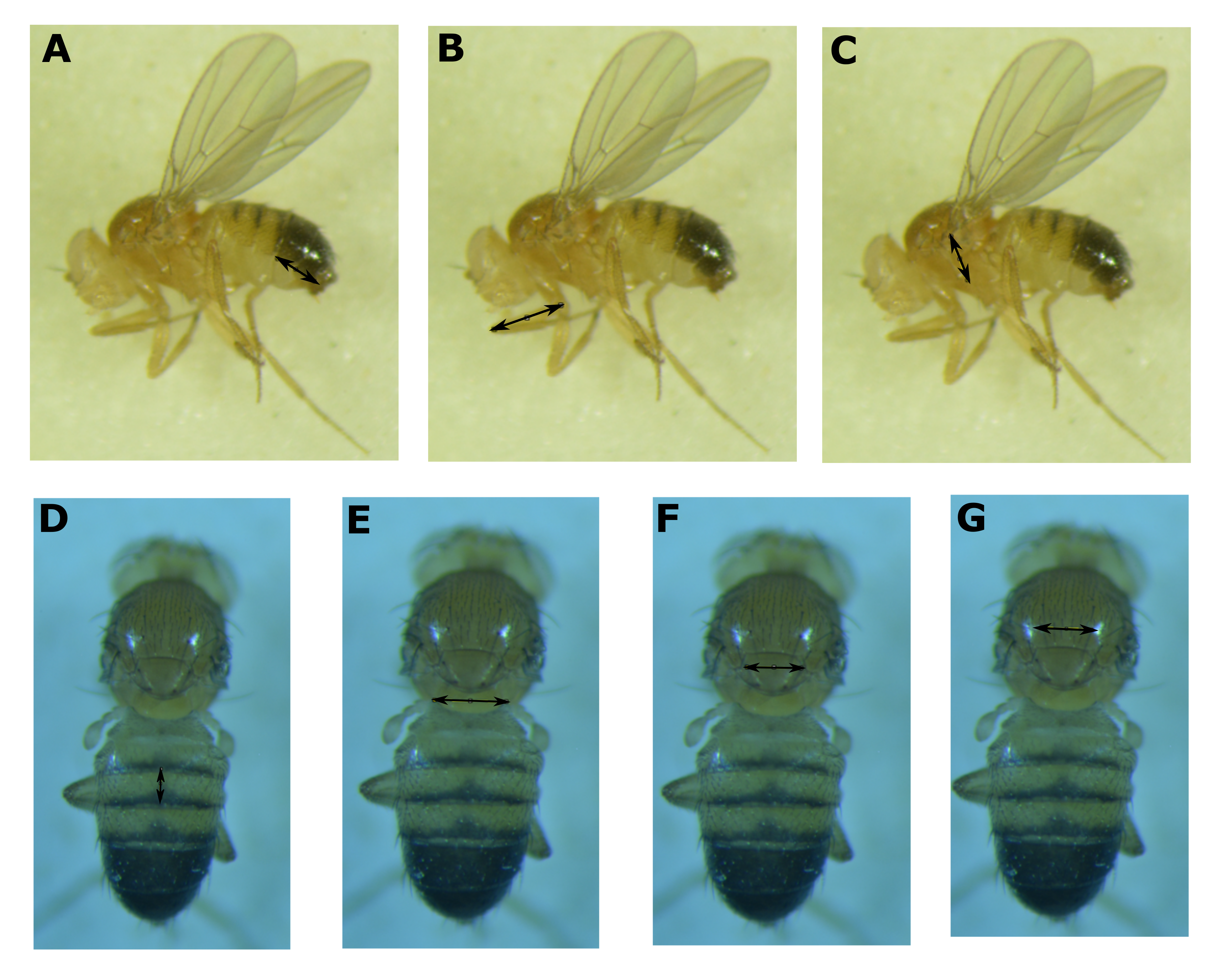

Supplement: S7 Fig — Measurements taken between the arrows using ImageJ. (TIF) [file pgen.1009297.s007.tif]
