# Supplementary material for: Dis3L2 regulates cell proliferation and tissue growth through a conserved mechanism
Source: PLoS Genet. 2020 Dec 28;16(12):e1009297. doi: 10.1371/journal.pgen.1009297 (PMC7793271; doi:10.1371/journal.pgen.1009297)
Supplement: S1 Table — (DOCX) [file pgen.1009297.s008.docx]

| Primer Pair Name | Forward | Reverse | Function |
| --- | --- | --- | --- |
| PC cloning | TATATACCGGTATGCCTTACCCTTTATATCCCG | AAATTTTCTAGACTAAATTTGTTCTTCCATCTTT | Amplify *dis3L2* CDS from cDNA |
| PA cloning | TATATACCGGTATGTCGGAAACGTCAAGCGTCA | AAATTTTCTAGACTAAATTTGTTCTTCCATCTTT | Amplify *dis3L2* CDS from cDNA |
| ND creation | GCTCGCGATTTGAATGACGCCGTTTCTATAG | CTATAGAAACGGCGTCATTCAAATCGCGAGC | Creating nuclease dead mutation. |
| hDIS3L2 cloning | TATATACCGGTATGAGCCATCCTGACTACAGGA | AAATTTTCTAGATCAGCTGGTGCTTGAGTCCTCG | Amplify human *DIS3L2* CDS from cDNA |
| Idgf2 cloning | CACGAGGCCGCATGAAGGCGTGGATCTGGTT | CGCACTAGTGTTTATAATTGATAATTGTT | Amplify *idgf2* from cDNA |
| gRNA | GTCGGAAACGTCAAGCGTCAACG | AAACCGTTGACGCTTGACGTTTCC | Guide RNA for CRISPR |
| PCR screening | GTCGGAAACGTCAAGCGTCAACG | GAGCCCGCTTCGACTTCTTT | PCR screening for CRISPR mutations |
| Mutant Sequencing | CGGTCGTACATGATGGGACT | GCAAATGCGAAAGTGGCTGA | DNA sequencing of potential mutants |

**S1 Table: Primers used in this study**

Primers above used for generation of *dis3L2^12^* and the creation of *UAS-Dis3L2^PA^*, *UAS-Dis3L2^PC^*, *UAS-Dis3L2^ND^*, *UAS-hDIS3L2* and *UAS-idgf2.*

| Name | ID | Forward | Probe | Reverse |
| --- | --- | --- | --- | --- |
| *rp49* | Dm02151827_g1 | N/A | N/A | N/A |
| *idgf1* | Dm01842859_g1 | N/A | N/A | N/A |
| *idgf2* | Dm01842858_g1 | N/A | N/A | N/A |
| *idgf3* | Dm01807767_g1 | N/A | N/A | N/A |
| *miple1* | Dm01795005_g1 | N/A | N/A | N/A |
| *cas* | Dm02152001_g1 | N/A | N/A | N/A |
| *syt4* | Dm02135116_m1 | N/A | N/A | N/A |
| *pre-idgf1* | custom | TGCTAATCTGATGCTGTCTCTGACA | CCAATGTGAACTCCACTTGTAAG | TGGTTAATAAAATAGTTCCTACTATATGTAAAAATAGGATAT |
| *pre-idgf2* | custom | GTAATTATACTTTAGTTTAATTATGACTTTAGGGTACT | CAGAGCGGGAATATCA | ACAAAGTCCACCAGGCCATT |
| *pre-idgf3* | Custom | CAGTCTCACCGTTTTGCCAAAT | CTTACAGCTGGAGTTCAC | ACCAGATCGTCGGAATAAACAAAGTA |

Primers above used for qRT-PCR where assay ID is provided for commercially available primer/probes.
