# Supplementary material for: Dis3L2 regulates cell proliferation and tissue growth through a conserved mechanism
Source: PLoS Genet. 2020 Dec 28;16(12):e1009297. doi: 10.1371/journal.pgen.1009297 (PMC7793271; doi:10.1371/journal.pgen.1009297)

**S1 File: Additional RNA-seq information**

Summary of read counts and alignments for each RNA-seq replicate. Reads the FlyBase *Drosophila melanogaster* genome (r6.18) using HiSat2 v2.1.0

|  | Replicate | Paired reads | Reads passing filtering | Reads mapped |
| --- | --- | --- | --- | --- |
| *dis3L2^wt^* | 1 | 40640720 | 40287088 | 35223001 |
|  | 2 | 38263277 | 38000207 | 32528177 |
|  | 3 | 37614770 | 37366876 | 31096714 |
| *dis3L2^12^* | 1 | 43538281 | 43265493 | 36918445 |
|  | 2 | 36517562 | 36255393 | 31092625 |
|  | 3 | 39654701 | 39402096 | 33385395 |

Non-default parameters used for RNA-seq alignment and quantification.

| Program | Non-default parameters | Effect |
| --- | --- | --- |
| HiSat2 | -i 20 | Minimum intron size 20bp (default 70bp). |
|  | -I 150000 | Maximum intron size (default 500000). |
|  | -Cufflinks | Adds a tag used by Cufflinks in downstream analysis |
| Cufflinks | -u | More accurate weighting of reads mapping to multiple locations. |
|  | -N | Normalisation to upper quartile of number of fragments mapping to a locus instead of total number of fragments. Improves robustness of differential expression for lowly expressed transcripts. |
|  | -compatible-hits-norm | Only fragments compatible with reference transcript counted in FPKM calculation. |
|  | -M | Supplied a rRNA.gtf file to remove rRNAs from downstream analysis. |
| Cuffquant | -u | More accurate weighting of reads mapping to multiple locations. |
| Cuffdiff | -u | More accurate weighting of reads mapping to multiple locations. |

Comparison between replicates within each genotype


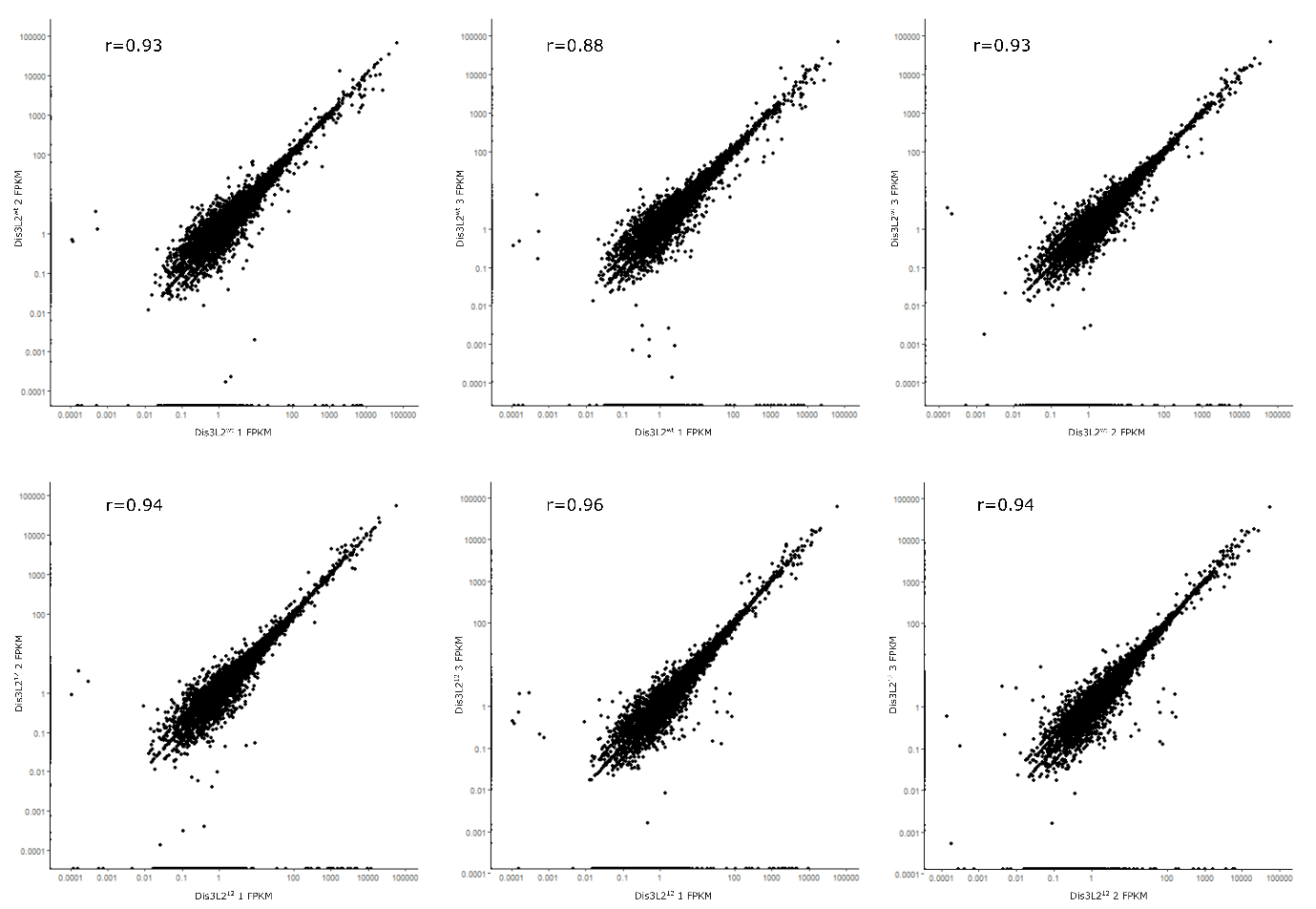

Supplement: S1 File — (DOCX) [file pgen.1009297.s009.docx]
