# Supplementary material for: Dis3L2 regulates cell proliferation and tissue growth through a conserved mechanism
Source: PLoS Genet. 2020 Dec 28;16(12):e1009297. doi: 10.1371/journal.pgen.1009297 (PMC7793271; doi:10.1371/journal.pgen.1009297)

**S2 File: Uncropped Western blots**

Fig 1B


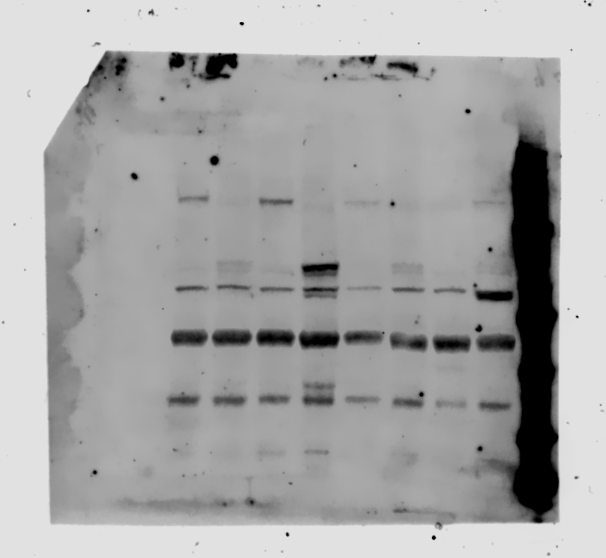


Fig 1H


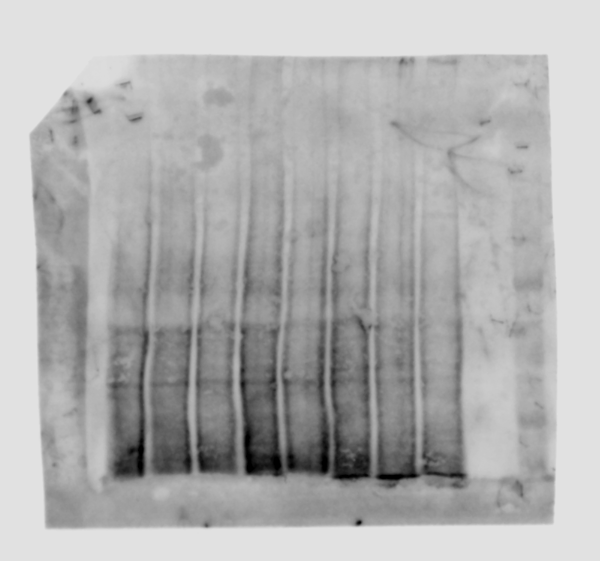


Fig 2A


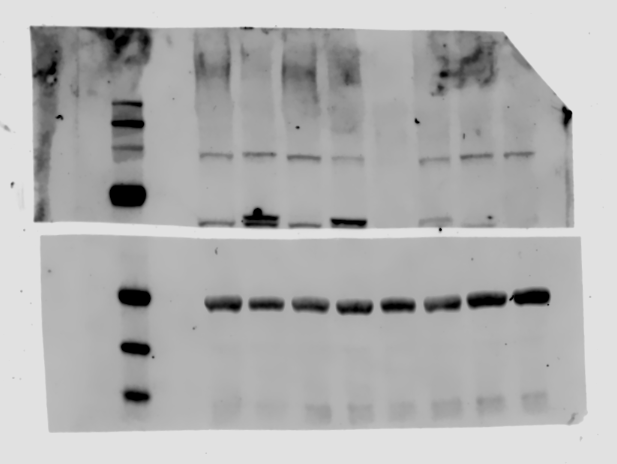


Fig 3A


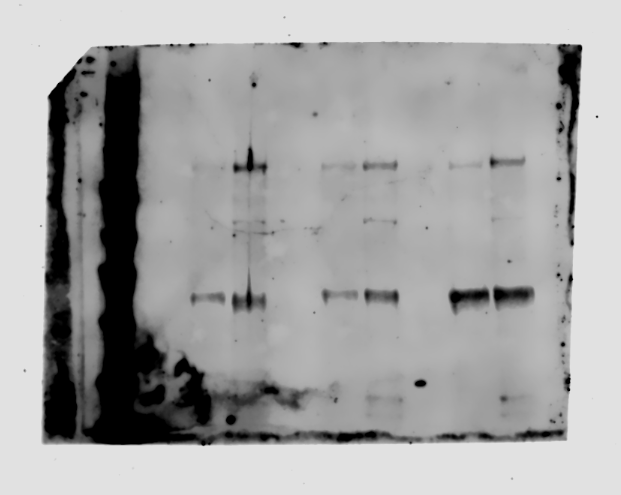


Fig 6C


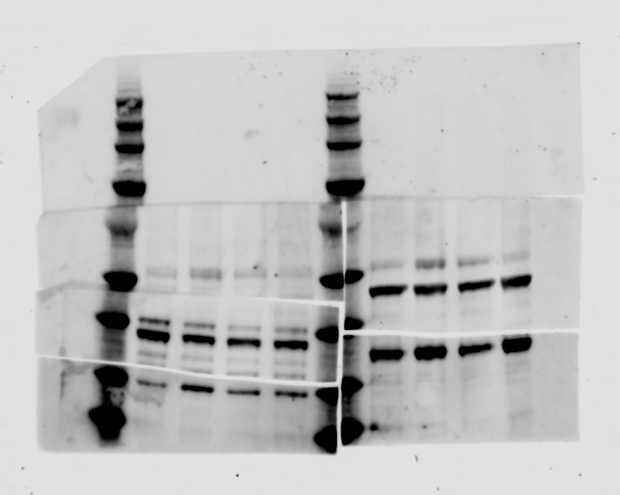

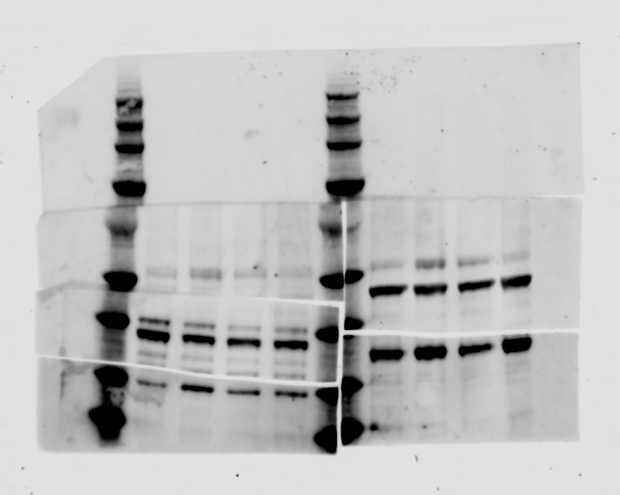


Fig 6D

S65 T70 T37/46


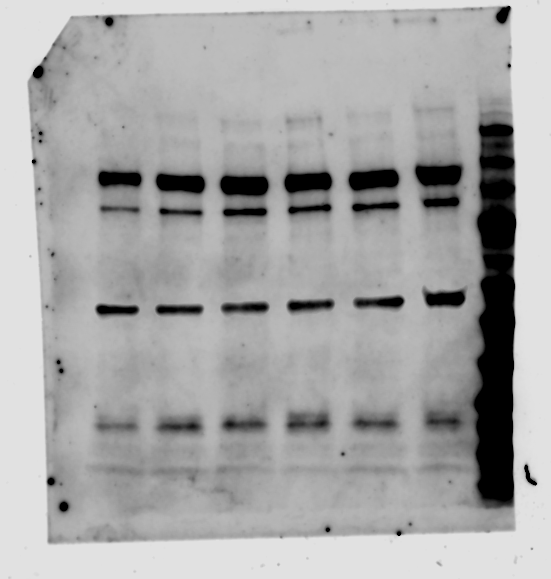

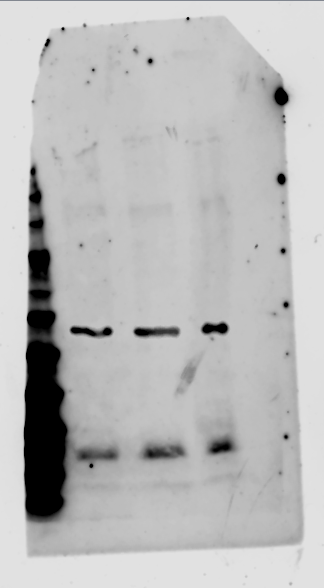

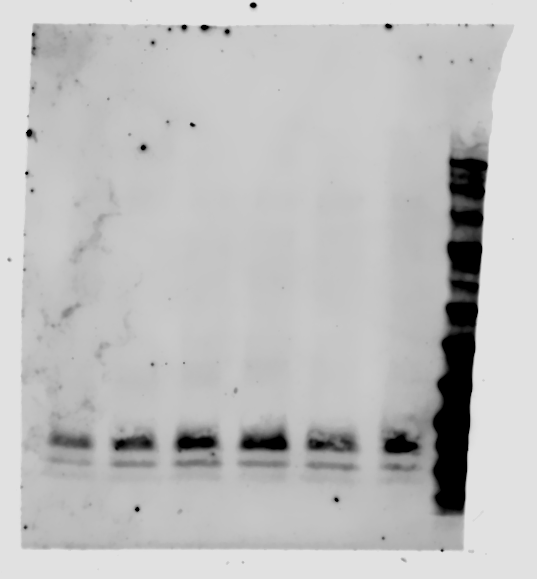


Fig 6F


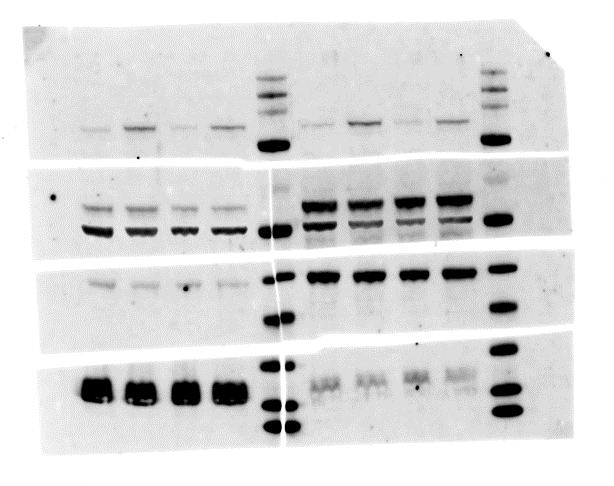


Fig 7E

Top Panel


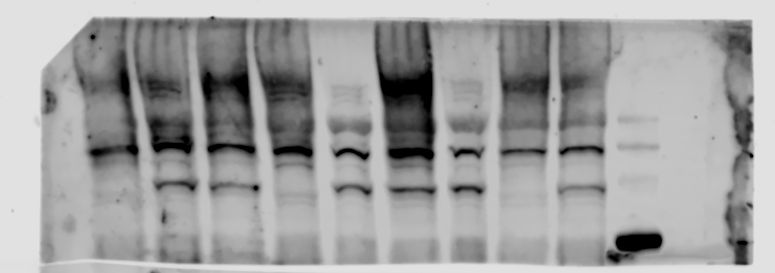


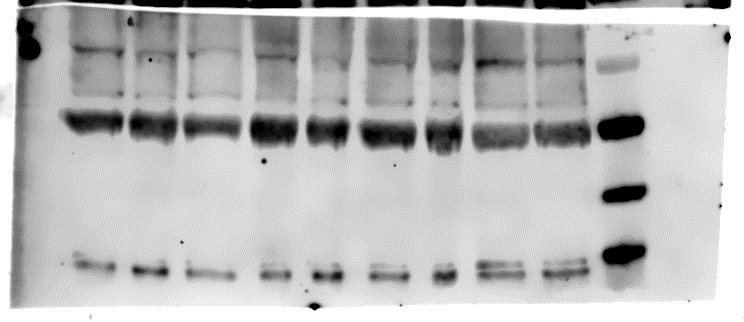


Bottom Panel


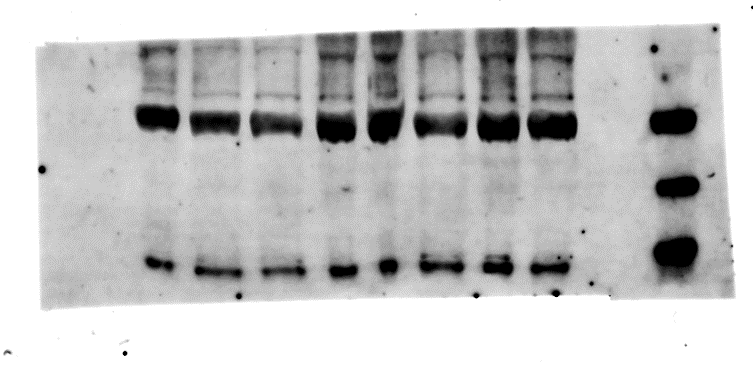


Sup Fig 2F


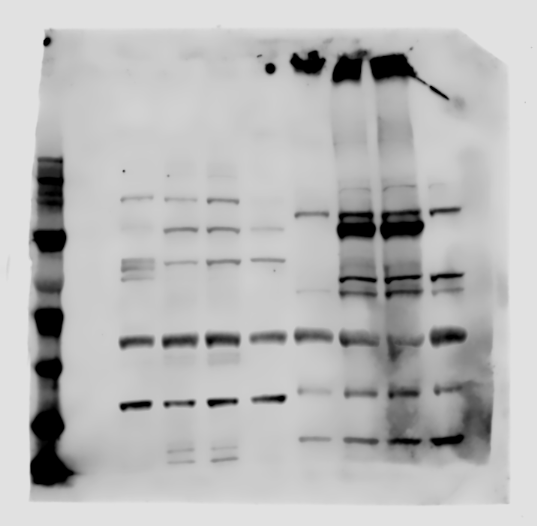


Sup fig 4A


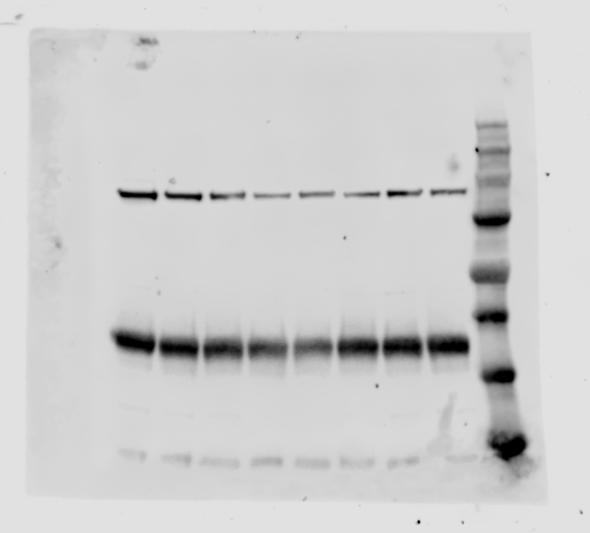


Sup Fig 6A


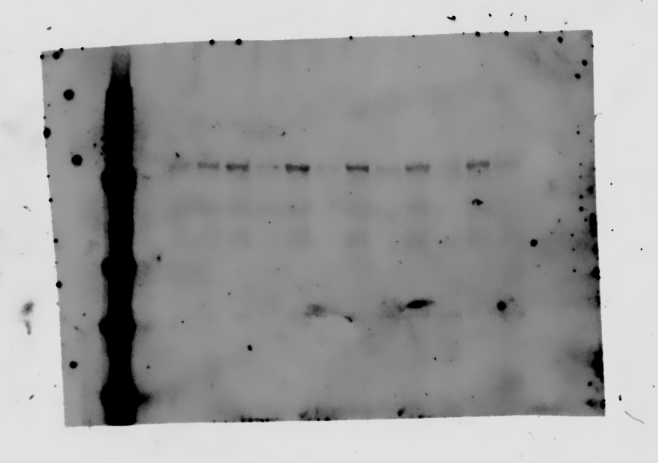


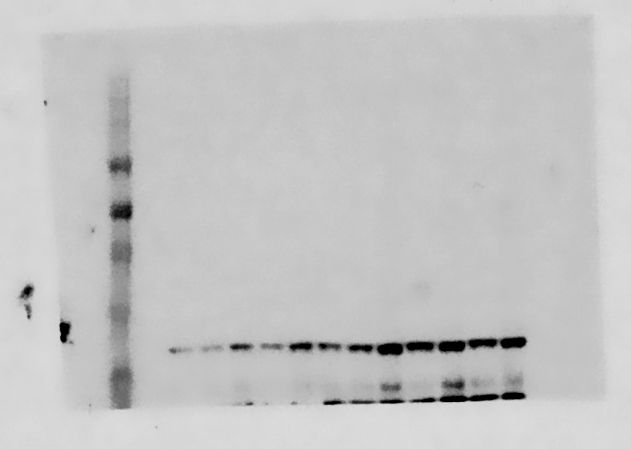


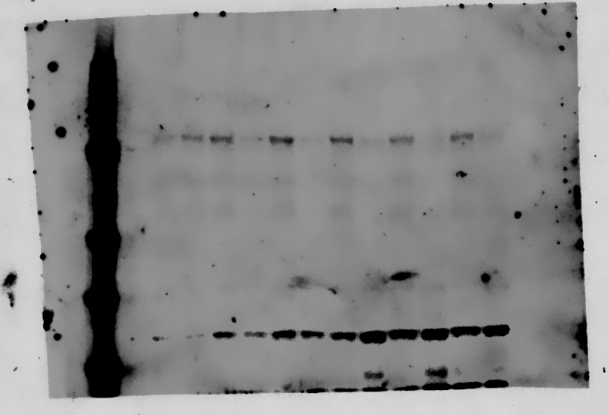


Sup Fig 6B


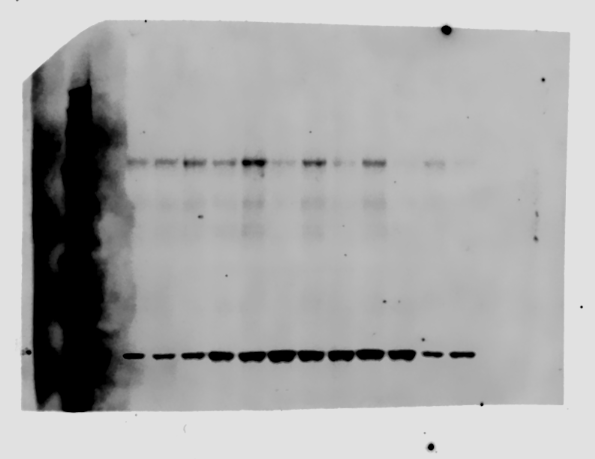


Sup Fig 6C


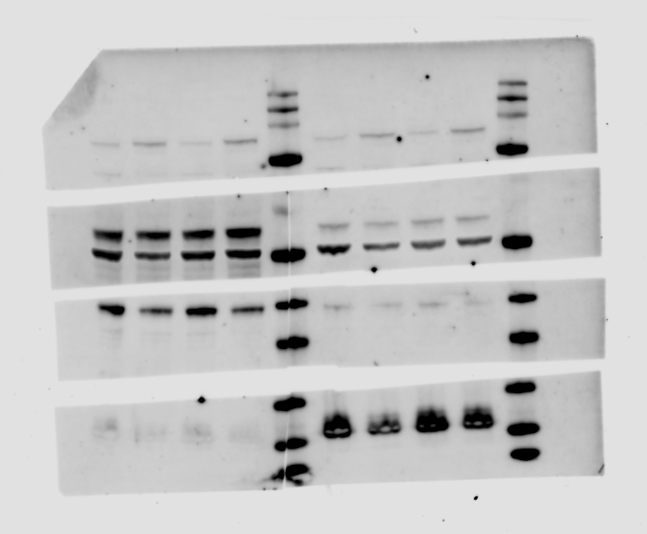


Sup Fig 6D


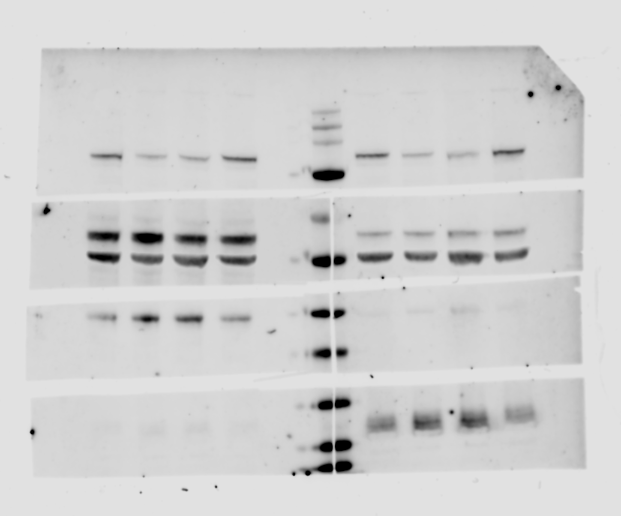


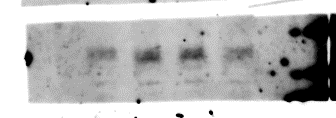

Supplement: S2 File — (DOCX) [file pgen.1009297.s010.docx]
