# Supplementary material for: Dis3L2 regulates cell proliferation and tissue growth through a conserved mechanism
Source: PLoS Genet. 2020 Dec 28;16(12):e1009297. doi: 10.1371/journal.pgen.1009297 (PMC7793271; doi:10.1371/journal.pgen.1009297)
Supplement: Attachment — Submitted filename: Response_to_Reviewers.pdf [file pgen.1009297.s011.pdf]

Reply to referees

Thank you for your email giving us the opportunity to revise and re-submit our manuscript entitled "Dis3L2 regulates cellular proliferation through a PI3-Kinase dependent signalling pathway" (PGENETICS-D-20-00279).

We would like to thank the reviewers for taking the time to make their comments. We have provided a substantial amount of extra supporting data where requested by the referees and are pleased to say that we have been able to answer all of the referees comments (noted in blue font below). I hope that these revisions result in a paper suitable for publication in PLOS Genetics.

Reviewer #1:

In this paper Towler et al show that fly CRISPR mutants for the exoribonuclease Dis3L2 show overgrowth phenotypes in imaginal tissues and final adult organ size. Similarly, they find that knockdown of Dis3L2 in HEK293 cells also causes an increase in proliferation. Using a combination of RNA seq and analyses of published

Overall I thought that this was a good paper, showing the characterization of a new growth regulator. Most of my comments should be easy to address

1. The authors use a nice combination of cell culture and Drosophila experiments to ask how loss of Dis3L2 promotes overgrowth. The pinpoint upregulation of Idgf2 and upregulation of PI3K signaling as mechanisms. But it's not entirely clear whether these two effects are related. I think a couple more analyses could be done:
a) Does loss of Dis3L2 lead to upregulation of the homolog of Idgf2 in the HEK293 cells? If not, then it would suggest that the Idgf2 and PI3K/Akt mechanisms are independent.

We have attempted to assess levels of Idgf2 homologues in *DIS3L2* knockdown HEK-293T cells (see Figure 1 below). *CHI3L1* does show an increase in expression; however, the expression of both *CHI3L1* and *L2* is very low and their CTs are very high (~38) are therefore we are not confident enough in the data to include this within the manuscript. Our data confirms previous experiments where *CHI3L1* and *CHI3L2* have been shown to be expressed at very low levels in immortalised cell lines. We have made these points clear within the discussion section of the manuscript lines 392-395.

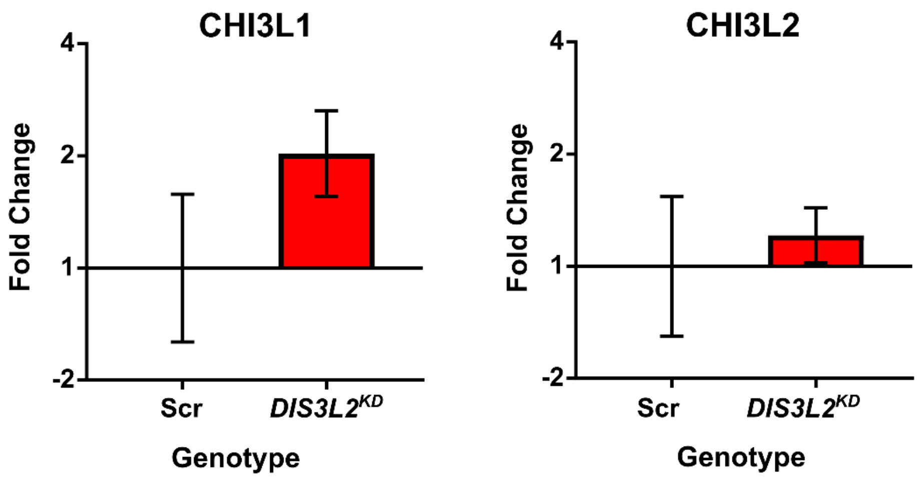

Figure 1: Assessing *CHI3L1/L2* expression in *DIS3L2* knockdown HEK-293T cells. Levels of *CHI3L1* and *CHI3L2* were assessed by qRT-PCR on HEK-293T cells 72 hours post transfection with wither siDIS3L2 (*DIS3L2*^{KD}) or siScrablmed control (Scr). Whilst a 2-fold increase in *CHI3L1* is detected the expression of both transcripts were very low and therefore confidence in the data is limited. n=4, error bars represent SEM.

b) Does loss of Dis3L2/overexpression of Idgf2 in Drosophila lead to increased Pi3K/Akt activity? This can be tested by looking at phospho-Akt western blots on discs or by using qRT-PCR to look at FOXO target genes in discs (these would be decreased with increased PI3k/Akt signaling). The wortmanin data indicate that Pi3K is required for Dis3L2/idgf2 regulated growth, but this may not mean that either or both actually regulate the pathway. If Pi3k/Akt activity isnt increased then it woudl again suggest idgf2 and Pi3k work in parallel, independent pathways to control growth.

We have now used Western blotting to assess phosphorylation of AKT on T342 (homologous to T308). *Drosophila* imaginal discs are very small and it is time consuming to dissect enough material for Western blotting for phospho-targets. We therefore performed the experiments on whole L3 larvae, which were subjected to accurate developmental timing using standard egg laying procedures. We demonstrate that there is an increase in pAKT T342 signal in *dis3L2*^{12} larvae compared to *dis3L2*^{wt}. We also observe increased phosphorylation of T342 in whole L3 larvae with ubiquitous Idgf2 overexpression (driven by *Tubulin-GAL4*). This would indeed suggest that the PI3-K pathway is activated in response to loss of Dis3L2/overexpression of Idgf2 and that they are not in a parallel pathway. This data has been added into Figure 7 and is discussed on lines 336-339.

The regulation of FOXO targets in response to PI3-K/AKT signalling is dependent upon phosphorylation of S505 (homologous to human S493). We do not see a consistent increase in S493 in HEK-293T cells (Figure 6) and therefore FOXO targets may not be downregulated.

2. Other than the imaginal tissues, are any other larval tissues increases in growth (e.g. fat

body, salivary gland) or is overall body growth/development increased. These are all sensitive to PI3K signaling, but it could be that Dis3L2 expression and function is limited to specific tissues, which would be interesting.

In line with the reviewers' suggestion we have assessed L3 larval mass and size in addition to salivary gland area. Unlike the imaginal tissues or adult fly size we observed no significant difference between Dis3L2 control and mutant animals. Therefore, this suggests that only the imaginal tissues are susceptible to overgrowth following the loss of Dis3L2. This is also consistent with an imaginal disc growth factor (Idgf2) being responsible to driving the observed overgrowth in Dis3L2 deficient animals. This data has been added into Sup Fig 1 and on lines 124-126

Reviewer #2: In this MS, Towler and cols study the role of Dis3L2 in growth control. They use the imaginal tissues of Drosophila as a primary model to determine the mechanisms used by this exoribonucease in the regulation of tissue size. The authors identify Idgf2 and PI3K signaling as elements mediating the growth regulatory role of Dis3L2. Growth control is a central aspect in biology and therefore this study is potentially interesting. However, the study is very preliminary and is not suitable for publication in Plos Genetics in its current form. Some issues need to be addressed.

Specific comments are:

It would be helpful showing in Fig 1 schematic representation of the dis3L2 gene indicating the position and nature of the dis3L2-12 mutation.

We have adapted Figure 1 to show a schematic representation of the mutation in panel A.

The authors should introduce the SuNSET labeling approach in the text. This would facilitate the understanding of this experiment.

In line with the reviewers' suggestion we have introduced SuNSET labelling prior to discussing the data on lines 127-132.

Line 147, the authors mention that act-Gal4 allowed expression of Dis3L2 to levels consistent with that of the control line. What do the authors mean with this? In the Fig is very clear that the levels are reduced, as compared to the control, which I guess is the +-line. The authors should clarify this.

We have now added the quantification level for each construct relative to the *dis3L2*^{wt} control into Sup Fig 2A and explained this within the manuscript on lines 144-151.

What is the phenotype of over-expressing dis3L2 in the wing? It should be shown in Fig 2.

In Figure 3 we demonstrate that strong over-expression Dis3L2 within the wing results in reduced growth and proliferation. However, in the rescue experiments, which were designed to show that the phenotypes observed were due to disruption of Dis3L2 and not another gene, we generated lines that only show a mild ~2 fold over-expression in *dis3L2*^{wt} animals (quantification data now shown in Sup Fig 2A and mentioned on line 148). Using

these constructs to induce mild ectopic expression of Dis3L2 with *nub-GAL4* results in a minor (4%) reduction in wing area (Sup Fig 2C) whilst no change in posterior area is observed when driven by *en-GAL4* (Sup Fig 2D). Therefore, the specific rescue observed in Figure 2 is not simply due to inappropriate ectopic Dis3L2 overexpression. This has been made clear within the text (lines 158-160) and the data added to Sup Fig 2.

What are the protein levels obtained when expressing the line that lacks catalytic activity? This should be shown In the western shown in Fig 2a, as well as the levels obtained when expressing the human protein. Otherwise, the results obtained are difficult to interpret and it is difficult to compare between the different conditions analyzed.

The *UAS-Dis3L2ND* line was created by using the same targeted insertion site that we used to generate the wild-type rescue lines. Therefore, the expression should be consistent with the expression of the wild-type constructs. This has now been confirmed by Western blotting and is now included in Figure 2A with the quantification in ectopic and rescue flies also included in Sup Fig 2A. We have also added an explanatory sentence in the text for clarity (Lines 165-167)

The levels obtained when expressing the human protein is shown in Sup Fig 2F. Due to the use of a different antibody to the human DIS3L2 protein we are unable to directly compare the levels to those obtained when driving the *Drosophila* constructs. To control for the expression of the human protein we used the same attP target site (i.e. the same genomic location) used for the generation of the *UAS-Dis3L2^{PC}*, *UAS-Dis3L2^{PA}* and *UAS-Dis3L2ND* lines therefore ensuring that similar amounts of RNA are expressed.

The authors suggest that different rescue obtained at different temperatures could be due to the human protein being more stable at 37C. It is worth noting that, generally, Gal4 driver are stronger at 29C than at 25C. I would suggest to consider and mention that in the text as a potential explanation.

In line with the reviewers' suggestion we have included this within the text on lines 176-177.

The authors use the anterior compartment as an internal control in the overexpression experiments showed in Fig 3. However, compartments accommodate in some cases to defects in growth (see PMID, 21179433). To prevent a misinterpretation due to potential final size effects mediated by accommodation, and to be consistent with previous Figs, would suggest to also use nub-Gal4 to perform this analysis. This comment also applies to Fig 5.

The reviewer is correct in that tissue compartments do communicate and can accommodate changes in growth rates in other regions of the tissue. However, the phenotypes/rescue in phenotype (Figures 3 and 5 respectively) that we observe are specific and therefore show the use of the driver is suitable. We are of the opinion that using a driver such as *en-GAL4*, which allows for the use for the anterior area as an internal control is preferable as one does not have to take culture conditions into account. For consistency we have now added *en-GAL4* driven Dis3L2 rescue data into Sup Fig 2C, which is consistent with our *nub-GAL4* driven data. We have also included representative images of control and Dis3L2

overexpression (*en-GAL4* driven) wings and wing imaginal discs into Figure 3 to aid the visualisation of the specific size effects.

As in Fig 1, representative images of adult wings should be provided in the Figure.

We have included representative images of adult wings in both Figure 2 and Figure 3. We have also included representative images and a diagrammatic overlay of the wing imaginal discs in Figure 3C'.

Why then the authors switch to a different Gal4 driver (69B) to study PH3? The authors should stick to the use of the same genetic tools to make the results obtained comparable. Using PH3 as a proxy of cell proliferation is not sufficient to conclude that cells proliferate less. PH3 labels cells in mitosis and, if mitosis occurs faster, the number of PH3 positive cells will be reduced with a similar final mitotic rate. Besides, discs of different ages have different proliferation rates and slight deviation in the timing of those discs can have a strong impact in the result obtained. The authors should use alternative tools as EdU incorporation as well as induce clones and calculate the cell doubling time (see PMID, 9657151) in the different conditions analyzed to present these results in a convincing manner. These analysis should also be performed in the loss of function of Dis3L2 and in a context of Idgf2 over-expression.

We switched to using *69B-GAL4* to study the effects of Dis3L2 and Idgf2 overexpression on proliferation as it drives expression throughout the wing imaginal disc. This facilitates accurate quantification of the number of the PH3 positive cells when using the DeadEasy MitoGlia plugin in ImageJ, which would not be possible if we were using compartment specific drivers such as *en-GAL4*.

PH3 staining is a standard method to assess the proliferation rate in *in-vivo* tissue and we have previously published the use of this method to demonstrate increased proliferation in Dis3L2 knockdown wing imaginal discs (driven by *69B-GAL4*, Towler *et al* 2016 doi: 10.1080/15476286.2016.1232238). Further, PH3 staining minimises the exposure to stress of the imaginal discs and does not depend upon consistent uptake rates as is the case with Edu/BrdU incorporation experiments. We have now performed PH3 staining in *dis3L2*^{wt} and *dis3L2*^{12} imaginal discs and, consistent with our previously published work, Dis3L2 deficient imaginal discs show an increased mitotic index. This data has been added to Figure 1 and is mentioned on lines 122-124.

We used restrictive 1hr egg lay periods to ensure that wing imaginal discs were age controlled; this has been made clear in the methods (line 591). To ensure that the PH3 staining is representative of the number of cells in the adult wing, we have calculated the number of cells in Dis3L2 null, Dis3L2 overexpression and Idgf2 overexpression adult wings, where Dis3L2 and Idgf2 overexpression was also driven by 69B-GAL4. As with the mitotic index calculations these analyses demonstrated an increase in cell number in Dis3L2 null and Idgf2 overexpression wings and a reduction in cell number in Dis3L2 overexpression wings. This data has been added to Figures 1, 3 and 5 and discussed on lines 117-119, 192-193 and 274-275 respectively.

The authors should validate by qPCR the top 30 genes shown in Fig 4. This would provide information about the sensitivity of the approach.

At this stage we do not have the funds to validate the top 30 genes. The successful validation of the 5 transcripts presented shows that we can have confidence in the RNA-sequencing data.

To link dis3L2 with PI3-K in the control of cell proliferation in the wing disc (Fig 7), the authors should analyze whether the manipulation of dis3L2 and Idgf2 (GOF and LOF experiments) results in changes in PI3K activity.

We have now performed Western blotting to assess activation of AKT on T342 (homologous to T308). As explained in the response to Referee 1 above, we have performed these experiments on whole L3 larvae. This demonstrates an increase in pAKT T342 signal in *dis3L2*^{12} larvae compared to *dis3L2*^{wt}. We also observe increased phosphorylation of T342 in whole L3 larvae with ubiquitous Idgf2 overexpression (driven by *Tubulin-GAL4*). This is congruent with our Wortmannin data suggesting that loss of Dis3L2 results in increased phosphorylation of AKT on T342 (which is catalysed by PI3-K) which subsequently drives overgrowth. This data has been added into Figure 7 and is discussed on lines 336-339.

Reviewer #3: Review of Towler et al. "Dis3L2 regulates cellular proliferation through a PI3-K dependent signaling pathway"

In this manuscript, Towler et al. show that loss of the conserved 3'-5' exoribonuclease Dis3L2 leads to increased cellular proliferation and overgrowth in Drosophila wing imaginal discs and HEK-293T cells. The authors present evidence that increased proliferation is due in part to PI3K activation, as wortmannin treatment reduces organ size and mitotic index in Dis3L2 mutant fly wings and cell number in HEK-293T cells with Dis3L2 knockdown. The investigators identified transcripts affected by loss of Dis3L2 by RNA sequencing of control and Dis3L2 mutant wing discs. This led to the identification of imaginal disc growth factors – Idgf1, Idgf2 and Idgf3 – as possible targets of the exoribonuclease activity of Dis3L2. Epistasis experiments to knockdown each Idgf individually in Dis3L2 mutants, generated by the authors using CRISPR/Cas9, show that Idgf2 is an important positive regulator of growth in wing discs lacking Dis3L2.

Overall, this study illustrates the importance of RNA processing as a contributor to cell and organ growth. The study would be strengthened by the addition of additional experiments that would shore up the authors' findings and connect Idgf2 with Dis3L2 and PI3K activation.

Major points.

Tissue growth.

1. Tissue- or compartment-specific manipulations of Dis3L2 or Idgf2 alter growth in a tissue-autonomous manner, as shown in Figures 2, 3 and 5. Do the authors find tissue-nonautonomous growth regulation by Dis3L2? For example, are leg lengths still increased in whole-animal Dis3L2 mutants that have wing-specific re-expression of wild type Dis3L2? This should be examined because a secreted factor – Idgf2 – accounts for growth

phenotypes in Dis3L2 mutants. Similarly, does overexpression of Idgf2 using 69B-GAL4 induce growth only in wings?

We have measured adult size in the specific *nub-GAL4* driven rescue animals and observe that they are still significantly larger than controls. This would suggest that the increased expression of Idgf2 acts in an auto or paracrine manner. This is also supported by the fact that we see a specific rescue in the posterior area of the wing following Idgf2 depletion in a *dis3L2* mutant background (Figure 5). The data has been added into Sup Fig 2B and discussed on line 153.

2. Changes in wing cell number in response to Dis3L2 and Idgf2 manipulations are inferred by assessing mitotic index (Figures 3D and 5E). The authors should determine whether these manipulations also alter cell size, as might be expected if PI3K is activated. Direct measures of cell number in wild type and Dis3L2 adult wings should be provided.

We have performed the suggested experiments on control and Dis3L2 null wings and have included the data in Figure 1 and in the text on lines 117-119. This shows that the primary effect is a change in cell number (10% increase). However, we do see a minor, although significant, 2% increase in cell size.

We have also analysed changes in cell number and cell size in relation to Dis3L2 overexpression when driven by *69B-GAL4*. This demonstrated a reduction in the total number of cells in the wing, consistent with the mitotic index calculations shown in Figure 3D. This data has been added to Figure 3 and discussed in the text on lines 192-193.

Finally, we have also assessed the number and size of wing cells in response to Idgf2 overexpression when driven by *69B-GAL4*. Consistent with our hypothesis, this revealed a very similar effect to loss of Dis3L2 where the change in area is predominantly driven by a change in cell number but also a small increase in cell size. This data has been added into Figure 5 and mentioned on lines 274-275.

RNA-sequencing.
3. The authors used flies with a 6-nt deletion of Dis3L2 as a control for their RNA-sequencing. Data should be provided in a supplement showing that this allele is wild type with regard to animal size, wing growth, and Dis3L2 protein expression.

The 6nt deletion does not affect the expression of Dis3L2 or the wing area or either males or females compared to $dis3L2^{wt}$ controls. This data has been included in Sup Fig 4A.

4. RNA sequencing data suggest that Dis3L2 regulates transcripts with a shorter than average 3’-UTR. The authors find enrichment of a U-rich motif in the 3’-UTR of Dis3L2-regulated transcripts but absence of a G-rich motif. Are any of these features found in the Idgf2 3’-UTR?

The 3’ UTR of Idgf2 is very small at 50bp in length and does not contain G rich motif as expected. Although there is no extended U-rich region, there is an AU-rich element which may be involved in targeting to Dis3L2. This has now been discussed on lines 457-459.

Dis3L2 and PI3K/mTOR signaling.
5. The connection between Dis3L2, Idgf2, and PI3K activity is inferred from results showing

that wortmannin feeding partially rescues wing size in Dis3L2 mutants and in flies overexpressing Idg2 in wing. The authors should use immunocytochemistry for tGPH, a PIP3 reporter, or for phosphorylated Akt to determine whether insulin signaling is stimulated by compartment-specific Dis3L2 knockdown or Idgf2 overexpression. Wortmannin can also inhibit mTORC1, so it is particularly important to see whether the upstream part of the PI3K-mTORC1 pathway is induced by loss of Dis3L2 and inhbited by wortmannin feeding.

We have now performed Western blotting on control, Dis3L2 null or Idgf2 overexpression larvae to assess AKT phosphorylation on T342 in line with the suggestions of other reviewers. This has demonstrated an increase in T342 phosphorylation in both Dis3L2 null and Idgf2 overexpression animals demonstrating that the upstream part of the PI3-K-mTORC1 pathway is indeed activated in Dis3L2 null animals and therefore showing pathway conservation between *Drosophila* and human kidney cells. This data has been added into Figure 7 and is discussed on lines 336-339.

6. Signaling experiments in HEK-293T cells are uninterpretable without additional controls. First, total levels of the phosphorylated kinases and substrates measured in Figure 6 and Supplemental Figure 6 must be shown. This is especially important because the authors describe that the PI3K/Akt pathway is significantly differentially expressed in HEK-293T cells lacking Dis3L2 (although direction of change is not specified). It is possible that an increase in basal levels is driving the pathway, rather than autocrine signaling.

We have now assessed the levels of total AKT, PRAS40 and 4E-BP expression in $DIS3L2^{KD}$ HEK-293T cells. We observe no significant increase in the expression of AKT or PRAS40 and only see a small (25%) increase in 4E-BP levels. This therefore suggests that autocrine signalling is indeed behind the overgrowth phenotype, although perhaps a feed-forward or parallel pathway is also influencing the basal levels of at least 4E-BP. This data has been included in Sup Fig 6C and on lines 311-313.

Second, data in Figure 6F showing rescue of elevated phosphorylation of Akt and TORC1 targets in wortmannin-treated Dis3L2 knockdown HEK-293T cells are impossible to interpret without vehicle controls on the same blot. The experiment should be repeated. Vehicle controls are also needed for the 48h and 72h time points shown in Supplemental Figure 6C. Third, phospho-Thr308 Akt should be sensitive to wortmannin treatment as well. This should be assessed.

In line with the reviewers' suggestion we have repeated these experiments in the presence of the vehicle control and included pAKT at T308 on the blots at 48 and 72hrs post transfection. This data has been added into Figure 6F and Sup Fig 6D.

Data presentation.

7. Data should be presented as absolute measurements where possible. See, for example, Figure 1 where growth measurements are presented as percent increases over wild type rather than mm2 for disc or wing area or mg body weight. The percent increase can be described in the text, as it is currently.

Where possible we have now changed the figures to represent absolute measurements in line with the reviewers' suggestion.

8. Exact sample sizes (or a range) for each set of data should be reported in Figure Legends, rather than "n ≥ 22", for example.

We have changed the sample size in each legend to represent the range.

Discussion points.

9. The results from immortalized cell lines should be interpreted more carefully. HEK-293T cells are not a physiological model of kidney, and differences between HEK-293T cells and U2OS cells may not reflect tissue of origin, but genetic differences acquired as a result of immortalization or tumorigenesis.

In the discussion this possibility along with the genetic differences are mentioned. We have now expanded this section to add emphasis on the likelihood of this (lines 404-410).

10. Pre-mRNA levels of Idgf2 are increased by Dis3L2. As Dis3L2 is thought to act post-transcriptionally, this suggests a secondary effect caused by loss of Dis3L2. Furthermore, wortmannin treatment of Dis3L2 mutants partially rescues wing size without altering Idgf2 mRNA levels. The authors should comment on these discrepancies in the Discussion.

The data we present suggests both a direct effect and indirect effect on *idgf2* expression. For, example, the CLIP data clearly suggests that Dis3L2 directly degrades *idgf2*. We have added this into the discussion on lines 362-365.

The data showing that *idgf2* mRNA levels are elevated in *dis3L2* mutant tissues shows that Idgf2 lies upstream of the wortmannin-sensitive pathway consistent with a role for Idgf2 is driving proliferation in a wortmannin-sensitive manner as demonstrated in Figure 7D. This has been discussed and clarified on lines 340-342.

11. The authors discuss the two activating phosphorylation sites on Akt and their relative contributions to Akt activity (lines 458-467). Indeed, a number of studies show that phosphorylation of the activation loop threonine (308 in mice/342 in flies) is critical for Akt activity, and that phosphorylation of the hydrophobic motif serine (473 in mice/505 in flies) is important for full activity and control of a subset of substrates. These papers should be cited. See: Cheng et al., JCB, 2011; Guertin et al., Dev Cell, 2006; Hietakangas and Cohen, G&D, 2007; Jacinto et al., Cell, 2006; Kumar et al., Diabetes 2010; Lee and Chung, BBRC, 2007.

These references have been included within the manuscript and discussed on lines 420-424.

Minor points.
1. Careful proofreading of the manuscript is necessary. A few examples of errors to correct are:
Defining abbreviations (line 77), grammar (lines 182-184, 406, 436-440), subject/verb agreement (lines 189, 272-273, 494), spelling (line 349)

We thank the referee for highlighting these errors. They have now been corrected.

2. Molecular weights should be indicated on Western blots.

Molecular weights have been provided on all Western blots.

3. Scale bars should be added to Figures 1A, 3C', and S7.

Scale bars have been added to all figures containing representative wing or wing imaginal disc images.

4. The legend for Supplementary Figure 1D should be written to specify the genotype of the female flies used in these crosses.

This legend has been edited to state that *dis3L2*^{wt} virgin females were used for all the crosses to assess male fertility.

5. The human transgene data from Figure 2B should be plotted with the data in 2C. As presented, it is not obvious that the 29˚C data in 2C show a rescue compared with the 25˚C data in 2B.

This data was kept separate as wing area at two different temperatures cannot be compared because wing area reduces in size as the temperature increases. Therefore, it is not possible to compare the data at 29°C to that at 25°C. We have changed the axis to represent mm^2 and therefore hope that this is clearer.

6. In Figure 3, the authors use an EP insertion line to show that Dis3L2 overexpression in wing disc compartments reduces compartment size. Do the UAS-Dis3L2 transgenes that were generated for this study also reduce compartment size when expressed under control of en-GAL4 or 69B-GAL4? Also: representative images of wings for B and C should be included, either in the main text or as a supplemental figure.

As mentioned in response to reviewer 2, driving the new transgenes with *nub-GAL4* results in a very mild 4% reduction in wing area whilst driving these lines with *en-GAL4* does not have an effect on the posterior area of the wing suggesting that the mild ~2 fold overexpression in these conditions does not have a strong effect on wing area. This data has been included in Sup Fig 2 and mentioned on lines 158-160.

7. The log scale in Figure S4A obscures the differences in 3'-UTR length between transcripts misexpressed in Dis3L2 mutants compared with controls. Also, is this difference in 3'-UTR length statistically significant?

The change in 3' UTR length is statistically significant and this has now been included within the text on line 207 and in the legend of Sup Fig 4. We feel that changing the figure from a log scale would result in a figure that is not intelligible and therefore have not made this change. We hope that the inclusion of UTR lengths and the statistical information helps the reader to understand the changes.

8. What is the GA4 driver used for Figure 5B?

The *en-GAL4* driver was used as in Figure 5A. This has been added to the figure legend for clarity.

9. In Figure S5B, the authors show that re-expression of Dis3L2 in dis3L2 mutant wing discs reduces Idgf2 mRNA. Data for controls – dis3L2 mutants alone – should be added for comparison.

The data for the parental mutant lines (*UAS-Dis3L2*^{PC} *; dis3L2*^{12}) has been added to the figure for comparison.

10. Cell types should be indicated in the legend for Supplemental Figure 6, panels A and B.

We have added the cell types into the legend for Sup Fig 6A and B.
