# Supplementary material for: Dis3L2 regulates cell proliferation and tissue growth through a conserved mechanism
Source: PLoS Genet. 2020 Dec 28;16(12):e1009297. doi: 10.1371/journal.pgen.1009297 (PMC7793271; doi:10.1371/journal.pgen.1009297)
Supplement: Attachment — Submitted filename: Response_to_Reviewers.pdf [file pgen.1009297.s012.pdf]

We would like to thank the reviewers for their time in considering our manuscript. We are gratified that all three reviewers believe the new data has significantly improved our manuscript and thank them for their suggestions. Given the remaining concerns of reviewer 3 we have adjusted our title and the strengths of our conclusions regarding that nature of PI3-Kinase pathway upregulation. We include individual responses to the remaining minor concerns below. The changes made during this round of revisions are in blue text within the manuscript. We hope that these additional changes make our manuscript suitable for publication in PLoS Genetics.

Reviewer #1: The authors have addressed all my comments and I think the paper is ready for publication

We are gratified that the reviewer believes that our paper is ready for publication. We have replied to and addressed the minor comments below.

I only had a couple of minor comments:

1. in the methods, the authors described doing western blots on imaginal discs. Did they include these results in the paper? I think the new pAkt data from Fig 7E on whole larvae would be bolstered by complementary blots on discs (if these expts were done).

As outlined in our initial response we were unable to get pAKT blots working sufficiently well on imaginal discs due to the small tissue size. The imaginal disc blots were performed on the *en-GAL4* driven Dis3L2 overexpression experiments displayed in Fig 3A. We are confident that the system-wide increase in pAKT together with the rescue of wing area following wortmannin treatment suggests that pAKT increase in the larvae is at least partially driven by changes in the imaginal discs. However, we have added a sentence on p16 lines 384-389 to state the limitations of the work on whole larvae rather than the wing imaginal discs themselves.

2. Many blots are heavily cropped with only a thin sliver of each blot with the relevant bands being shown. In these cases, I think its always useful to have images of the uncropped versions as supplemental.

We have included a new supplemental file containing the uncropped image for each Western blot performed. The region shown in the figure is highlighted with a black box.

Reviewer #2: The authors have assessed all my concerns and the paper is ready for publication in it current estate.

We are very pleased that we have addressed the reviewers concerns and that they believe the study is now suitable for publication.

Reviewer #3: In the revised manuscript, “Dis3L2 regulates cellular proliferation through a PI3-Kinase dependent signaling pathway", Towler et al. provide new data that strongly support their findings of an overgrowth phenotype in Drosophila wing imaginal discs lacking Dis3L2 and convincingly show that the growth factor Idgf2 is a strong driver of growth in Dis3L2 mutant tissue. In particular, very nice, new data show that the overgrowth phenotype of Dis3L2 mutants is largely due to an increase in cell number, with only a 2% increase in

cell size.

We are pleased, and fully agree with the reviewer that the new data does indeed strengthen our conclusions upon the mechanism behind Dis3L2-regulated growth in *Drosophila*.

Statistical tests are lacking for these data:
Sup Figure 1D
Sup Figure 2C – no statistics are shown comparing compartment areas, although the text refers to significant differences or lack of differences (lines 157-158 and 167-170).
Figures 6A, 6B and 6E

We apologise for missing these statistical analyses in the previous versions of the manuscript. We have added the relevant statistical information to the figures and in the figure legend. Statistical analyses are difficult on lifespan/growth curves without high sample sizes, therefore on consultation with our Chartered Statistician at Brighton and Sussex Medical School (Dr Chris Jones), we have presented the graphs with 95% confidence intervals and determined significant differences by the lack of overlap between the error bars.

The authors' conclusion that Dis3L2 affects growth via PI3K signaling is not well supported by the data presented, as described below.

First, Western blots shown in Figures 6 and 7 do not include total levels of Akt, PRAS40, MAPK, 4E-BP, etc. for each sample. Second, the phosphorylation levels are normalized by comparing Dis3L2 mutant/knockdown animals/tissue to controls. Instead, levels of phospho-Akt, for example, should be normalized to levels of total Akt in each genotype, and this should be plotted. It is still unclear that apparent changes in phospho-protein levels are driven by activation of the pathway through signaling rather than by changes in total kinase or substrate levels.

We have now added the phosphorylated protein normalised to the total protein levels alongside the total protein blots in Sup Fig 6C and added this information on p13 lines 312-319. We have acknowledged the limitations of our data throughout the discussion to temper our claims. We also point out that further work is required to better understand the mechanism behind AKT, PRAS40 and 4E-BP phosphorylation and whether this is solely driven at the post-translational level or if it is a combination of transcriptional/translational and post-translational effects.

Second, wortmannin seems to reduce proliferation of HEK293 cells with Dis3L2 knockdown but has little effect on Akt or PRAS40 phosphorylation. A dose response curve would be helpful in showing that wortmannin effectively inhibits PI3K here.

We show in Figure 6F and Sup Fig 6C that wortmannin treatment reduces the level of pAKT, pPRAS40 and p4E-BP specifically in DIS3L2 knockdown HEK-293T cells which suggests that wortmannin is effectively inhibiting the targeted pathway.

Third, modest changes in phospho-Akt (Thr342) in whole Dis3L2 mutant larvae are presented as evidence that PI3K signaling is activated in these animals. Total Akt levels are

needed to interpret this data, but another problem is that whole-animal changes in Akt may reflect systemic endocrine changes in Dis3L2 mutants. Given the authors' work with imaginal discs, I proposed that they measure either phospho-Akt or tGPH using immunofluorescence in discs. This could be done in a compartment specific manner, and it would help to address whether changes in Akt phosphorylation might occur tissue- or cell-autonomously in response to Dis3L2 or Idgf2 manipulations.

As outlined in response to reviewer 1 above we were unable to get sufficient signal to measure pAKT directly in the wing imaginal discs. We have added some text on p16 lines 384-389 to address the limitations of using whole larvae which indeed show a system wide effect. However, we are confident that the data demonstrating rescue of wing area following wortmannin treatment suggests that pAKT increase in the larvae is at least partially driven by changes in the imaginal discs.

We were unable to get an antibody to total AKT working well enough in *Drosophila* and therefore are unable to normalise to the total AKT levels. We have addressed these limitations within the text p14/15 lines 345-347 and discussed how further work is required to identify the nature of increased activity of AKT within the fly.

Finally, as outlined the changes we observed are quite subtle which explain the ordered overgrowth that we observe within our mutant animals. Therefore, we do not believe immunofluorescence to be sufficiently quantitative to measure such changes. We did perform some preliminary experiments using the *en-GAL4* driver to compare anterior to posterior pAKT expression in the wing disc however, as expected, the quantification was extremely variable. We therefore we did not pursue this avenue any further and see western blotting as a superior approach.

At present, other possibilities, including parallel, independent growth pathways regulated by PI3K and Dis3L2, could explain the results presented here and therefore the title should be reconsidered.

In line with the reviewers suggestion we have changed the title of the paper and as outlined in our previous responses have adjusted the strengths of our conclusions within the text accordingly.
